# Estimation of Health and Economic Benefits of a Small Automatic External Defibrillator for Rapid Treatment of Sudden Cardiac Arrest (SMART): A Cost-Effectiveness Analysis

**DOI:** 10.3389/fcvm.2022.771679

**Published:** 2022-02-24

**Authors:** Marcus S. Shaker, Elissa M. Abrams, John Oppenheimer, Alexander G. Singer, Matthew Shaker, Daniel Fleck, Matthew Greenhawt, Evan Grove

**Affiliations:** ^1^Dartmouth Geisel School of Medicine, Hanover, NH, United States; ^2^Department of Medicine, Dartmouth-Hitchcock Medical Center, Lebanon, NH, United States; ^3^Department of Pediatrics, University of Manitoba, Winnipeg, CA, United States; ^4^Department of Medicine, Rutgers New Jersey Medical School, Newark, NJ, United States; ^5^Department of Family Medicine, University of Manitoba, Winnipeg, CA, United States; ^6^Altrix Medical, Centreville, VA, United States; ^7^Department of Computer Science, George Mason University, Fairfax, VA, United States; ^8^Department of Pediatrics, University of Colorado School of Medicine, Aurora, CO, United States; ^9^Heart and Vascular Center, Dartmouth-Hitchcock Medical Center, Lebanon, NH, United States

**Keywords:** sudden cardiac arrest, automated external defibrillator (AED), early defibrillation, survival, cost-effectiveness analysis

## Abstract

**Background:**

Sudden cardiac arrest (SCA) occurs in 0.4% of the general population and up to 6% or more of at-risk groups each year. Early CPR and defibrillation improves SCA outcomes but access to automatic external defibrillators (AEDs) remains limited.

**Methods:**

Markov models were used to evaluate the cost-effectiveness of a portable SMART (SMall AED for Rapid Treatment of SCA) approach to early SCA management over a life-time horizon in at-risk and not at-risk populations. Simulated patients (*n* = 600,000) who had not received an implantable cardioverter defibrillator (ICD) were randomized to a SMART device with CPR prompts or non-SMART approaches. Annual SCA risk was varied from 0.2 to 3.5%. Analysis was performed in a US economy from both societal (SP) and healthcare (HP) perspectives to evaluate the number of SCA fatalities prevented by SMART, and SMART cost-effectiveness at a threshold of $100,000/Quality Adjusted Life Year (QALY).

**Results:**

A SMART approach was cost-effective when annual SCA risk exceeded 1.51% (SP) and 1.62% (HP). The incremental cost-effectiveness ratios (ICER) were $95,251/QALY (SP) and $100,797/QALY (HP) at a 1.60% SCA annual risk. At a 3.5% annual SCA risk, SMART was highly cost-effective from both SP and HP [ICER: $53,925/QALY (SP), $59,672/QALY (HP)]. In microsimulation, SMART prevented 1,762 fatalities across risk strata (1.59% fatality relative risk reduction across groups). From a population perspective, SMART could prevent at least 109,839 SCA deaths in persons 45 years and older in the United States.

**Conclusions and Relevance:**

A SMART approach to SCA prophylaxis prevents fatalities and is cost-effective in patients at elevated SCA risk. The availability of a smart-phone enabled pocket-sized AED with CPR prompts has the potential to greatly improve population health and economic outcomes.

## Key Points

**Question**: Is a smart-phone enabled pocket AED cost-effective in decreasing the mortality and morbidity of sudden cardiac death and arrhythmia, which account for 15–20% of all deaths worldwide?

**Findings**: Using a smart-phone enabled pocket-size AED with CPR prompts is cost-effective in patients with an annual SCA risk >1.51–1.62% and may prevent upwards of 100,000 SCA deaths in the United States.

**Meaning:** Availability of a personal smart-phone enabled small AED with CPR prompts for rapid treatment of SCA has the potential to significantly improve population health and economic outcomes.

## Introduction

Every day, almost 1,000 people experience sudden cardiac arrest (SCA) in the United States with a staggering 90% fatality rate (1). From a societal perspective (SP), sudden cardiac death (SCD) in the United States results in an estimated 2 million years of potential life lost for men and 1.3 million years for women (2). In fact, estimated deaths attributed to SCD exceed all other individual causes of death, including lung cancer, accidents, chronic lower respiratory disease, cerebrovascular disease, diabetes mellitus, prostate cancer, and colorectal cancer (2). Worldwide, SCD and arrhythmia account for 15–20% of all deaths with the majority occurring in patients without cardiac risk factors (3). Cardiovascular disease is a leading cause of global mortality, accounting for almost 17 million deaths annually. In developing countries, it causes twice as many deaths as HIV, malaria and TB combined. It is estimated that about 40–50% of all cardiovascular deaths are SCDs, and about 80% of these are caused by arrhythmias (4–6).

**Figure 1:**
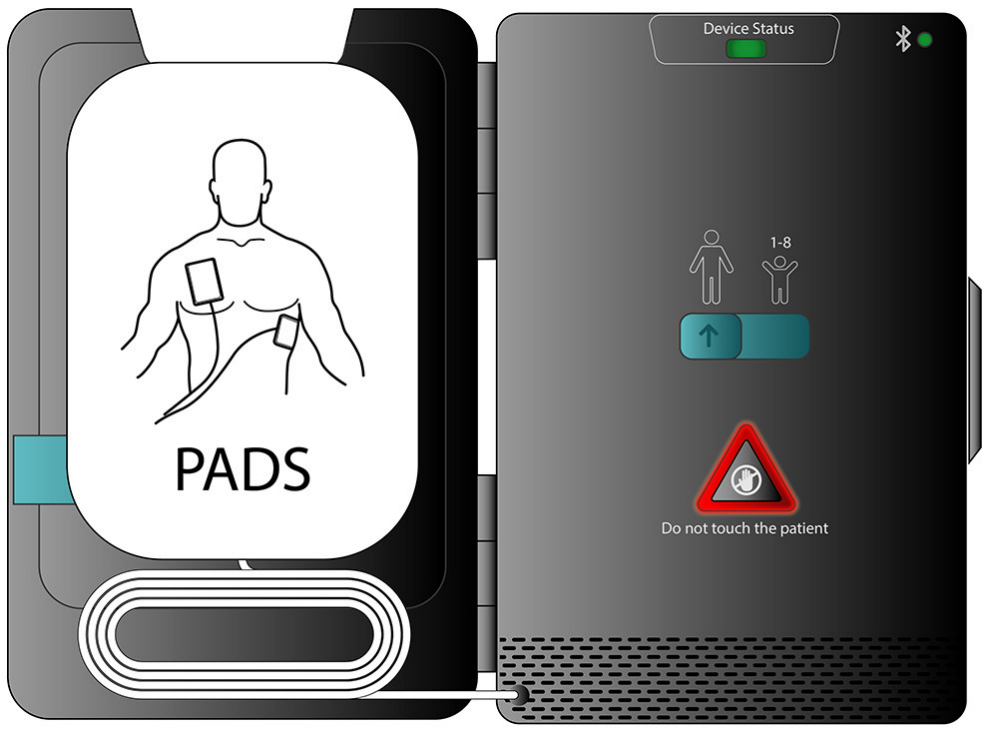
A smart phone enabled pocket AED. This SMall AED for Rapid Treatment of SCA (SMART) allows an approach to SCA prophylaxis that fosters rapid access to defibrillation.

Timely intervention with early cardiopulmonary resuscitation (CPR) and an automated external defibrillator (AED) greatly improves outcomes for SCA (7, 8). While AEDs are abundant in public locations and large population centers, these devices are poignantly largely unavailable where most SCAs occur, given nearly 70% of SCA occurs at home (1). Additionally, AEDs are large and may be cumbersome to easily transport, which has limited the ability to have an AED available at all times. To help solve the portability/transportability issue, recently, a smart phone enabled pocket AED has been developed. This SMall AED for Rapid Treatment of SCA (SMART) takes advantage of miniaturization to treat SCA in a novel way (Figure 1). The SMART device allows rapid access not only to effective CPR prompts, but also to early defibrillation.

In addition to device size, another reason AEDs may not be more available is that their cost (generally >$1,000) exceeds what individuals may wish to spend for personal use (9). However, considering cost separate from the device outcome fails to balance the tremendous benefit timely use of these devices can provide to persons at risk (1). Indeed, the ability to have an affordable and accessible, pocket-sized AED that can attach to a smartphone device has broad public health implications worldwide (4–6). The purpose of this study is to characterize the cost-effectiveness of the personal SMART device in populations at low, moderate, and high-risk for SCA.

## Methods

### Model Structure

**Figure 2:**
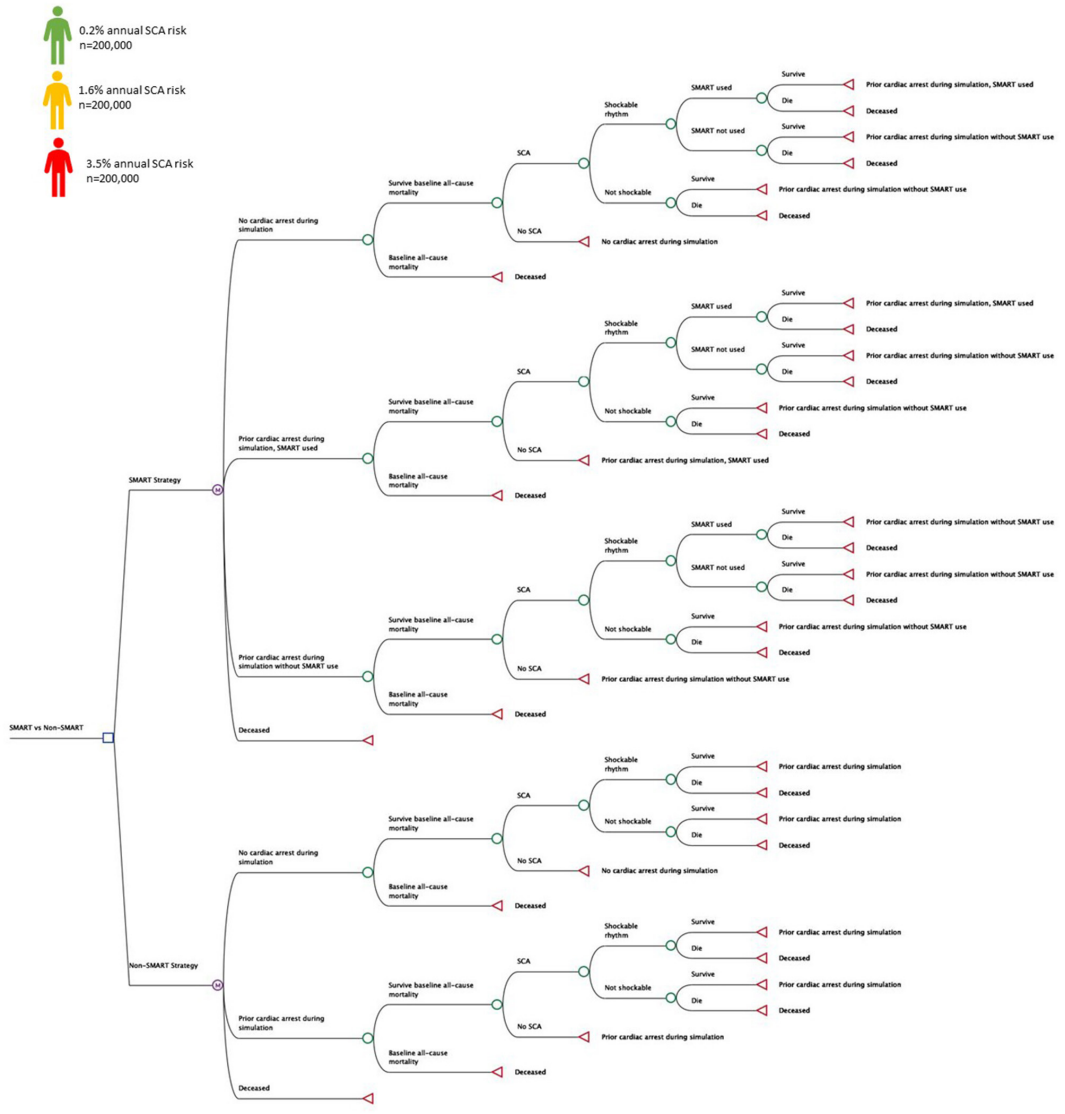
Decision Tree. Decision tree representing health states and transitions of a personal SMART (SMall AED for Rapid Treatment of SCA) approach vs. a non-SMART approach. Patients at varying risk for SCA (*n* = 600,000) were randomized to each approach during the simulations to evaluate SMART fatality reduction and cost-effectiveness.

TreeAge Pro 2021 (Williamstown, MA) is a decision analysis software package that was used to evaluate a SMART approach to prevent SCA morbidity and mortality compared with a non-SMART approach (Figure 2) (10). Markov models compared universal and risk-stratified SMART approaches using cohort and microsimulation approaches. Patients receiving implantable cardioverter defibrillators (ICDs) were not included in the analysis. Microsimulation tracker modifications were used to evaluate SMART fatality reduction on a population level, randomizing *n* = 600,000 patients across ranges of annual SCA risk (0.2–3.5%) (9, 11, 12). In addition to SCA risk, all patients assumed all-cause non-SCA mortality, estimated from United States Life Tables (13). The base case was represented by a 45 year old adult at risk for out of hospital SCA assuming an annual probability of SCA and associated morbidity and mortality. A 50-year model horizon was used to evaluate long-term outcomes with a cycle length of 1-year from both the healthcare (HP) and societal (SP) perspectives (14). From the SP, the modeling included health sector considerations with health outcomes (health related quality-of-life effects) and medical costs (paid for by third-party payers and by patients out-of-pocket) and non-health sector considerations including lost productivity and funeral cost savings for SCA deaths averted with SMART use (14). The analysis conducted from the HP excluded lost wages and funeral costs from preventable SCA deaths in those without SMART access (14). Health outcomes included fatalities prevented and incremental cost-effectiveness ratio (ICER) at threshold cost-effectiveness of $100,000 per quality-adjusted life year (QALY) (9, 15, 16). The analysis conformed to the Professional Society for Health Economics and Outcomes Research (ISPOR) Consolidated Health Economic Evaluation Reporting Standards (CHEERS) statement (17). Model inputs were identified from literature review and this simulation did not involve human subjects and was exempt from review by the Colorado Multiple Institution Review Board.

### Probabilities and Events

Table 1 outlines base-case assumptions. The PARAMEDIC trial was used to estimate likelihood of a shockable rhythm (ventricular fibrillation or ventricular tachycardia) of 23% and the probability of witnessed arrest by a family member or other person able to use SMART was estimated at 55% (22, 23). While survival rates of non-shockable arrest are 4.4–5.7% (12, 24), the tight linkage of quick access to CPR and early defibrillation increases survival for out-of-hospital ventricular-fibrillation SCA (1, 25–28). In a study of 200 patients living in Olmstead County Minnesota, with out-of-hospital ventricular fibrillation, Bunch et al. reported mean time from 911 call to first shock was 5.7 min (SD, 1.6 min) in survivors, and 6.6 min (SD, 1.5 min) in non-survivors (*p* = 0.002). In this study, 142 (72%) patients with ventricular fibrillation who received early defibrillation survived to hospital admission and 84 (42%) survived to hospital discharge (25). In a Japanese nationwide, prospective, population-based registry of out-of-hospital cardiac arrest by Kitamura et al., 49.6% of individuals with bystander-witnessed ventricular-fibrillation arrest survived and had return of spontaneous circulation before hospital arrival (with 44.7% 1-month survival) vs. 29.1% of those without public access to defibrillation (with 27.9% 1-month survival) (22). Not all patients suffering SCA receive early defibrillation, and in a prospective observational study of New York City 911 emergency response, the median on-scene dispatch to patient response time was 7.6 min (29). For modeling purposes, 49.6% of patients with ventricular-fibrillation arrest who used SMART were assumed to survive to hospital admission with a total survival rate to hospital discharge of 44.7% (22); those patients with ventricular-fibrillation arrest without SMART had survival to admission and discharge rates of 29.1% and 27.9%, respectively (22). Incremental benefit associated with the SMART CPR device prompts was incorporated into the overall survival benefit.

**Table 1:** Model assumptions.

**Description**	**Base case value**	**Sensitivity range**		**References**
Costs^*^				
SMART initial costs	$1,275	$800	$2,500	AED.US. (18)
Annual cost of pads (amortized)	$34	$10	$100	
Amortized annual cost of replacement AED	$128	$50	$300	
Annual battery cost (amortized)	$42	$30	$100	
Prehospital care cost	$1,134	$500	$2,000	Lurie (19)
Hospitalization cost for those admitted but not surviving to discharge	$9,282	$1,000	$15,000	
Hospitalization cost for those admitted and surviving to discharge	$39,475	$20,000	$50,000	
Annual wage	$57,764	$0	$57,764	US Bureau of Labor Statistics. (20)
Funeral cost	$9,000	$400	$15,000	(21)
**Probabilities**				
Annual SCA risk	0.2–3.5%	0.1%	6.0%	Cram et al. (9) Knops et al. (11)
Probability of moderate survivor impairment following VF SCA without SMART	11.0%	3%	15%	Kitamura et al. (22)
Probability of moderate survivor impairment following VF SCA with SMART	9.7%	1.7%	17.7%	
Probability of severe survivor impairment following VF SCA without SMART	12.6%	8.6%	16.6%	
Probability of severe impairment following VF SCA with SMART	5.9%	1.9%	9.9%	
Probability of no impairment following VF SCA without SMART	56.3%	46.3%	66.3%	
Probability of no impairment following VF SCA with SMART	77.5%	67.5%	87.5%	
Probability of coma following VF SCA without SMART	20.0%	14.0%	26.0%	
Probability of coma following VF SCA with SMART	6.9%	0.9%	12.9%	
Probability of no impairment in SCA survivors with non-shockable rhythm	22.9%	12.9%	32.9%	Lascarrou et al. (12)
Probability of moderate impairment in survivors SCA with non-shockable rhythm	12.5%	2.5%	22.5%	
Probability of severe impairment in survivors SCA with non-shockable rhythm	64.6%	54.6%	74.6%	
Probability of coma in survivors SCA with non-shockable rhythm	0%	0%	10%	
Probability of ventricular fibrillation or ventricular tachycardia in SCA	23.0%	15.0%	31.0%	Perkins et al. (23)
Probability SMART witnessed by family member or other person able to use device	55.0%	50.0%	67.0%	Kitamura et al. (22)
Probability of survival to hospital admission without SMART with VF arrest	29.1%	19.1%	39.1%	
Probability of survival to hospital admission with SMART with VF arrest	49.6%	39.6%	59.6%	
VF SCA overall survival without SMART	27.9%	17.9%	37.9%	
VF SCA overall survival with SMART	44.7%	40%	55%	
SCA survival without shockable rhythm	4.4%	2.4%	6.5%	Chan et al. (24)
**Utilities**				
HSU, no SCA	1.0	0.9	1	Cram et al. (9)
HSU, moderately impaired	0.2	0.15	0.5	
HSU, prior SCA and unimpaired	0.85	0.7	0.9	
HSU, severely impaired	0.1	0.05	0.4	
HSU, death or coma	0	–	–	
**Additional assumptions**				
Start age	45	40	75	
Discount rate	0.03	0	0.03	

* *Costs expressed in 2021 US dollars. VF, ventricular fibrillation. SCA, sudden cardiac arrest*.

### Costs

All costs were expressed in 2021 dollars with future dollars discounted at a rate of 3% per annum (Table 1) (30). The cost of SMART was based on initial costs and maintenance of the personal AED (18). Costs of prehospital and hospital care were adjusted for survival to hospital admission and discharge (19). The SP included additional funeral costs for SCA deaths not prevented by SMART and lost productivity of wage earners younger than 66 years of age, with the annual wage estimated from the United States Bureau of Labor Statistics occupational employment and wage statistics of all occupations (20, 21).

### Health State Utilities

Health state utilities (HSU) are measured on a 0–1 scale, with “1” representing perfect health and “0” representing death (31). Health state utilities represent patient preferences for competing health states measured under conditions of risk, and were used to derive quality adjusted life years (QALY), with future QALY discounted in accord with costs (30–32). Patients who survive SCA may experience moderate to severe impairments reflected by diminished HSU (9). In one survey of 729 SCA survivors, Nichol et al. used the Health Utilities Index Mark 3 system (HUI3) to assess quality of life and reported mean scores of 0.75 (SD, 0.33) and 0.74 (SD, 0.35) at 3 and 6 months following SCA (33). Health state utilities is directly related to time of resuscitation, with mean HUI3 scores of 0.81, 0.76, and 0.65 for those with resuscitation times of <2, 3–10, and >10 min, respectively (34). Still, individuals who receive prompt defibrillation are less likely to experience impairment (25). For example, Bunch et al. reported the health of 50 SCA survivors who had received early defibrillation, measured on the Medical Outcomes Study 36-Item Short-Form General Health Survey (SF-36) (25). Patients with rapid access to defibrillation had mean SF-36 scores similar to the general population (45, SD 11.1 vs. 50 for age-sex matched comparators). The model accounted for differential probabilities of post-SCA impairment (SMART HSU, 0.81; non-SMART HSU, 0.65) (34). In the study by Kitamura et al., significant differences in Cerebral Performance Category disability were reported for patients who survived ventricular-fibrillation arrest with prompt access to defibrillation compared to those without public access (good performance, 77.5 vs. 56.3%; moderate disability 9.7 vs. 11.0%; severe disability 5.9 vs. 12.6%; coma 6.9 vs. 20%) (22). Rates of disability for those surviving non-shockable arrests were based on Perkins et al. (good performance, 85.5%; moderate disability, 8.4%; severe disability, 5.6%; coma 0.6%) (23). Cram et al. reported persistent HSU of patients with prior SCA who were unimpaired, moderately impaired, and severely impaired of (0.85, 0.20, and 0.10, respectively), which were used for modeling purposes (9).

### Sensitivity Analyses

Deterministic sensitivity analyses were performed across plausible ranges (Table 1) for all variables. Additional analyses were performed excluding costs of a replacement AED device, at higher rates of SMART utilization, alternate ranges of SCA survival, with higher SCA hospitalization costs, and excluding differential rates of SMART post SCA impairment in HSU. Probabilistic sensitivity analyses (PSA) were performed to evaluate contemporaneous uncertainty of multiple variables and stochastic variation in variable assumptions using triangular distributions of modes and deterministic sensitivity ranges. Probabilistic sensitivity analyses were validated using alternative beta distributions for probabilities of healthcare utilization across annual SCA risk strata and gamma distributions for costs. For gamma distributions, standard deviations were set to one quarter of the mean value, with beta distributions modeled using the observed number of events (*r*) as the alpha and the at-risk population not experiencing the event as the beta. Further PSA validation was performed using alternate seeding.

## Results

### Cohort Analysis

A SMART strategy was cost-effective when the annual risk of SCA exceeded 1.51% (SP) and 1.62% (HP). At a 1.6% annual SCA risk, the ICER associated with a SMART strategy was $95,251/QALY (SP) and $100,797/QALY (HP). In populations with greater annual SCA risk, SMART was increasingly cost-effective. At a 3.5% SCA risk, SMART was associated with an ICER of $53,925/QALY (SP) and $59,672/QALY (HP) (Table 2).

**Table 2:** SMART cost-effectiveness.

**Cohort analysis**								**Microsimulation**		
**Strategy**	**Cost**	**Incremental Cost**	**Effectiveness (QALY)**	**Incremental effectiveness**	**ICER**	**NMB**	**C/E**	**Strategy**	**Fatality**	
									***Sum***	***Per patient SD***
**0.2% Risk for SCA (per annum) societal perspective**								**0.2% Risk for SCA**		
Not SMART	$2,106		20.66037			$2,063,931	$102	Not SMART (*n* = 1,000)	6,172	0.24
SMART	$7,503	$5,397	20.66857	0.00820	$657,886	$2,059,355	$363	SMART (*n* = 1,000)	6,046	0.24
**0.2% Risk for SCA (per annum) healthcare perspective**								*SMART SCA deaths prevented*	*126*	
Not SMART	$214		20.66037			$2,065,823	$10			
SMART	$5,655	$5,441	20.66857	0.00820	$663,226	$2,061,202	$274			
**1.6% Risk for SCA (per annum) societal perspective**								**1.6% Risk for SCA**		
Not SMART	$14,881		17.06670			$1,691,789	$872	Not SMART (*n* = 1,000)	39,607	0.49
SMART	$19,618	$4,737	17.11643	0.04973	$95,251	$1,692,025	$1,146	SMART (*n* = 1,000)	38,893	0.49
**1.6% Risk for SCA (per annum) healthcare perspective**								*SMART SCA deaths prevented*	*714*	
Not SMART	$1,439		17.06670			$1,705,231	$84			
SMART	$6,452	$5,013	17.11643	0.04973	$100,797	$1,705,191	$377			
**3.5% Risk for SCA (per annum) societal perspective**								**3.5% Risk for SCA**		
Not SMART	$27,858		13.55247			$1,327,389	$2,056	Not SMART (*n* = 1,000)	65,349	0.48
SMART	$31,990	$4,132	13.62909	0.07662	$53,925	$1,330,920	$2,347	SMART (*n* = 1,000)	64,427	0.48
**3.5% Risk for SCA (per annum) healthcare perspective**								*SMART SCA deaths prevented*	*922*	
Not SMART	$2,547		13.55247			$1,352,700	$188			
SMART	$7,119	$4,572	13.62909	0.07662	$59,672	$1,355,790	$522			

*QALY, quality-adjusted life year; ICER, incremental cost-effectiveness ratio; NMB, net-monetary benefit synthesis of QALY and costs at a conversion rate of $100,000 per QALY; C/E, cost-effectiveness*.

### Microsimulation

A SMART strategy demonstrated fatality reduction in microsimulation (cumulative SCA fatalities, per patient SD). Compared with a non-SMART approach, SMART prevented 1,762 deaths across 600,000 randomized patients. In the simulated patients with a 0.2% annual SCA risk (*n* = 200,000), 6,172 SCA deaths occurred in the non-SMART group (SD, 0.24) with 6,046 SCA deaths in the SMART group (SD, 0.24). For patients with an SCA annual risk of 1.6% (*n* = 200,000), 39,607 SCA deaths were recorded in the non-SMART group (SD, 0.49) with 38,893 deaths recorded in SMART patients (SD, 0.49). A SMART strategy prevented 922 SCA deaths for those patients with a 3.5% annual SCA risk (*n* = 200,000) (non-SMART: 65,349 deaths, SD 0.48; SMART: 64,427, SD 0.48) (Table 2). When considered from a larger United States population perspective, a SMART strategy could prevent an estimated minimum of 109,839 deaths in individuals 45 years and older (35, 36).

### Deterministic Sensitivity Analyses

**Figure 3:**
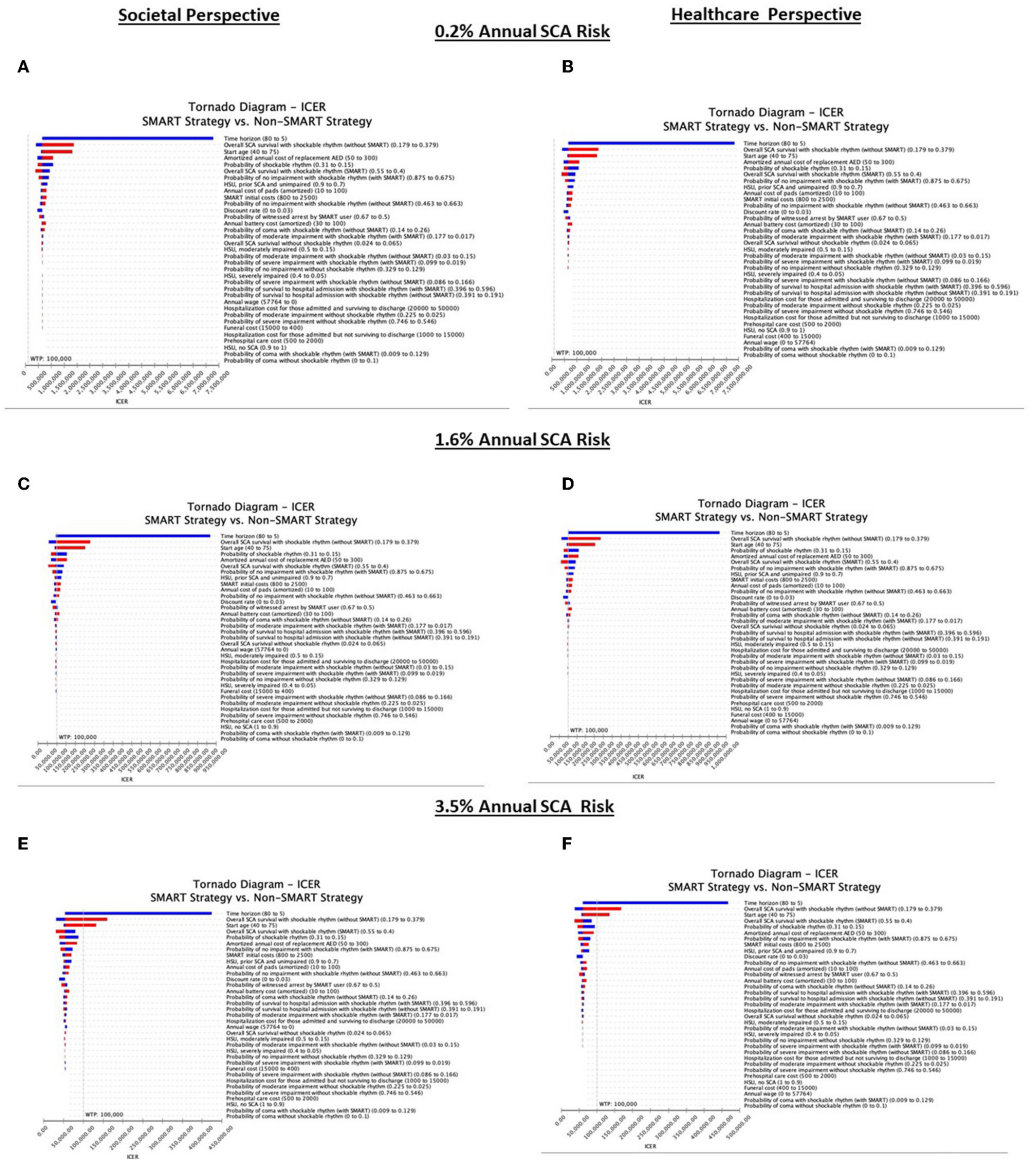
Deterministic sensitivity analysis. Deterministic analyses of patients at 0.2% annual SCA risk from the societal and healthcare perspectives **(A,B)**, patients at 1.6% annual SCA risk from the societal and healthcare perspectives **(C,D)**, and patients at 3.5% annual SCA risk from the societal and healthcare perspectives **(E,F)**. Cost-effectiveness is defined as care costing < $100,000 per QALY. Blue bars represent assumptions below the base case and red bars depict assumptions above the base case.

A SMART strategy was most sensitive to annual SCA risk (Figure 3). At an annual SCA risk of 0.2%, SMART was not cost-effective across any sensitivity threshold in either the SP or HP analysis. In contrast, at an annual SCA risk of 3.5%, SMART was cost-effective across all sensitivity ranges with the exception of time horizon [thresholds of <7.19 years (SP) and <7.25 years (HP)], overall SCA survival with shockable rhythm without SMART [thresholds of >34.70% (SP) and >34.20% (HP)], and start age [thresholds of >68.34 years (SP) and >67.95 years (HP)]. At an annual SCA risk of 1.6%, a SMART strategy was most sensitive to changes across multiple model inputs, including time horizon (threshold <7.48 years, SP) overall SCA survival with shockable rhythm without SMART (threshold >28.6%, SP), start age (threshold >47.72, SP), probability of a witnessed arrest by a SMART user and a shockable rhythm (thresholds <52.4%; <22.0%, SP), initial SMART costs (threshold >$1,512, SP), amortized annual cost of replacement AED, pads, and battery (thresholds >$142; $48; $56, SP), overall SCA survival with shockable rhythm using SMART (threshold <44.0%, SP), health state utility of prior SCA, unimpaired (threshold <81.9, SP), probability of no impairment with shockable rhythm with and without SMART (thresholds <75.8%; >59.6%, SP), probability of moderate impairment with shockable rhythm with SMART (threshold <2.3%, SP), and probability of coma with shockable rhythm without SMART (threshold >22.8%). In the SP analysis excluding funeral costs did not impact cost-effectiveness estimates (Figure 3). As rates of witnessed arrest fell, SMART became less cost-effective. For example, assuming only a 32% probability of witnessed arrest, at an annual SCA risk of 1.6%, SMART cost $163,322 (SP) and $168,870 (HP) per QALY.

**Figure 4:**
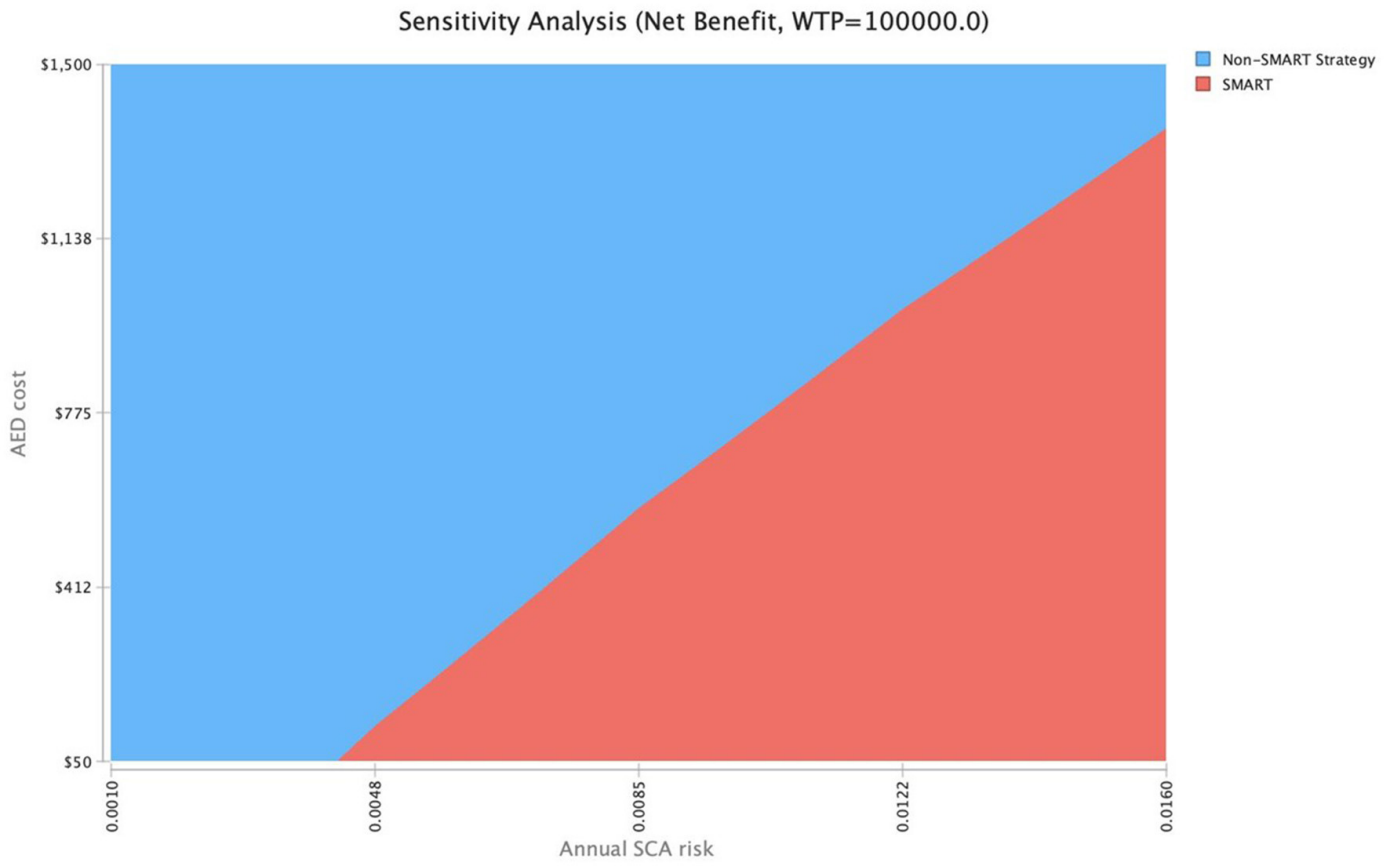
Two-way sensitivity analysis of AED cost and SCA risk. Assuming an $800 AED cost, a SMART approach would be cost-effective (WTP, $100,000/QALY) at a 1.0% SCA risk. At an AED cost of $488, a SMART approach would be cost-effective at a threshold SCA risk of 0.77%, and at an AED cost of $176, the SMART approach becomes cost-effective at an SCA threshold risk of 0.52%.

To further explore cost-effectiveness across ranges of SCA risk, a two-way sensitivity analysis was conducted to evaluate SMART cost-thresholds (Figure 4). Assuming an $800 AED cost, a SMART approach would be cost-effective [willingness to pay (WTP), $100,000/QALY] at a 1.0% SCA risk. At an AED cost of $488, a SMART approach would be cost-effective at a threshold SCA risk of 0.77%, and at an AED cost of $176, the SMART approach becomes cost-effective at an SCA threshold risk of 0.52%.

### Probabilistic Sensitivity Analyses

**Figure 5:**
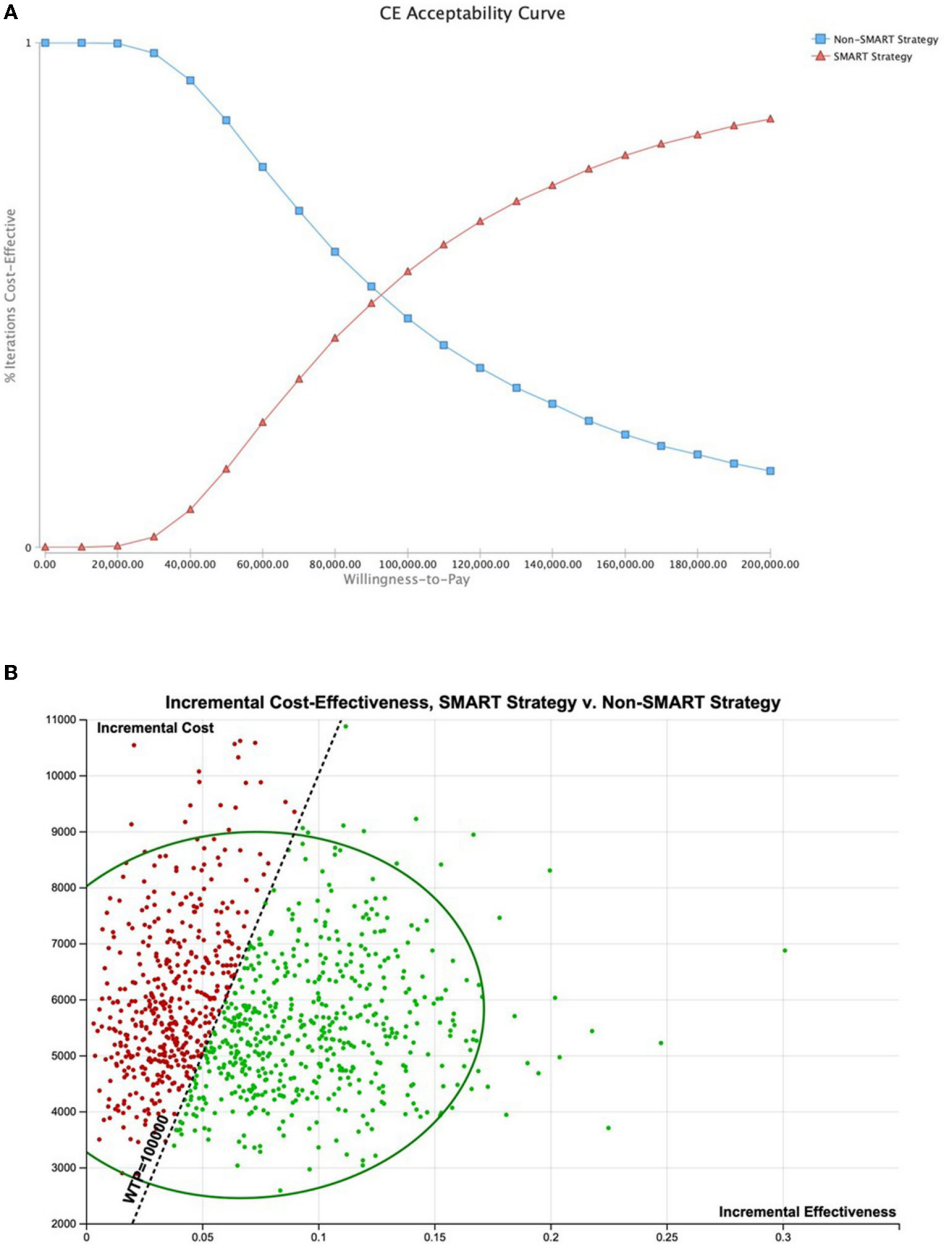
Probabilistic sensitivity analysis. Probabilistic sensitivity analysis using triangular distributions (*n* = 10,000 simulations, assuming an average 1.5% annual SCA risk with upper and lower limits of 0.1%–6.0%) demonstrated SMART to be the most cost-effective strategy in 54.64% of simulations (WTP, $100,000 per QALY). **(A)** Cost-effectiveness acceptability curve. **(B)** Cost-effectiveness of 10,000 simulations shown with 95% confidence ellipse.

Probabilistic sensitivity analysis (PSA) using triangular distributions (*n* = 10,000 simulations, assuming an average 1.5% annual SCA risk with upper and lower limits of 0.1%–6.0%) demonstrated SMART to be the most cost-effective strategy in 54.64% of simulations (WTP, $100,000 per QALY) (Figure 5). Use of alternative PSA seeding (*n* = 10,000 simulations) indicated SMART to be the most cost-effective strategy in 53.78% of simulations. In a population with a 5% annual SCA risk, SMART was optimal in 88.37% of simulations. Probabilistic sensitivity analysis using alternative distributions demonstrated SMART to be optimal in 76.71% of simulations for patients at this SCA risk.

## Discussion

Our analysis demonstrates a SMART strategy was cost-effective for patients with an annual SCA risk above 1.51% (SP) and 1.62% (HP). When considering SCA fatalities prevented over the 50-year simulation, SMART could save upwards of 100,000 lives in the United States by preventing death from SCA. While not cost-effective in patients with low annual SCA risk, a SMART strategy appears to provide attractive health and economic benefits in at-risk populations. For example, SCA risk varies by several clinical factors, including age, gender, race, total cholesterol, HDL, blood pressure, diabetes, and smoking status (Table 3) (37). Risk of SCA is a large consideration in SMART cost-effectiveness. For example, at a 1.6% annual SCA risk the SMART strategy is not cost-effective when the probability of a witnessed arrest falls below 52.4%; however, at a 3.5% annual SCA risk the SMART strategy would be cost effective even when the probability of witnessed arrest is only 29.3%.

**Table 3:** Estimates of atherosclerotic cardiovascular disease (ASCVD) risk.

**Gender**	**Age**	**Race**	**Total cholesterol**	**LDL cholesterol**	**HDL cholesterol**	**Treatment with Statin**	**Systolic BP**	**Treatment for hypertension**	**History of diabetes**	**Current smoker**	**Aspirin therapy**	**Baseline 10 years ASCVD risk (%)**	**Annual rate (%)**
M	45	Non-Hispanic White	165	80	45	N	115	N	N	N	N	1.40	0.14
M	50	Hispanic/Latino	175	95	55	N	120	N	N	N	N	2.30	0.23
M	45	African American	165	80	45	N	115	N	N	Y	N	5.70	0.59
M	50	Non-Hispanic White	210	140	25	N	130	N	N	N	N	7.80	0.81
F	55	Hispanic/Latino	255	160	35	Y	120	Y	Y	N	N	8.80	0.92
M	55	Non-Hispanic White	190	95	60	N	120	Y	Y	N	N	8.90	0.93
F	50	Asian/Pacific Islander—South Asian	200	135	25	N	140	N	N	Y	N	10.90	1.15
M	60	American Indian/Alaskan Native	140	90	40	N	130	N	N	Y	N	12.00	1.28
F	55	Hispanic/Latino	255	160	20	Y	120	Y	Y	N	N	14.60	1.58
F	55	Non-Hispanic White	210	140	20	N	140	N	N	Y	N	15.10	1.64
M	55	Non-Hispanic White	255	160	20	Y	120	Y	N	N	N	16.70	1.83
M	50	Asian/Pacific Islander—South Asian	200	135	25	N	140	N	N	Y	N	17.80	1.96
M	60	American Indian/Alaskan Native	140	90	20	N	120	Y	N	Y	N	19.50	2.17
M	60	Non-Hispanic White	260	160	30	N	150	N	N	N	N	20.10	2.24
M	55	Non-Hispanic White	230	150	30	N	140	N	N	Y	N	21.30	2.40
M	50	Non-Hispanic White	210	140	20	N	150	N	Y	N	N	22.70	2.57
M	55	Non-Hispanic White	210	140	20	N	140	N	N	Y	N	26.70	3.11

*Estimated from: American Heart Association (37). Green shading indicates cost-effective care for a SMART approach*.

These findings are consistent with prior evaluations of the economic impact of personal AEDs. Cram et al. evaluated the cost-effectiveness of in-home AEDs for individuals with standard and elevated risk for SCD (9). In their analysis, they concluded in-home AED use in all adults over 60 years of age was associated with an ICER of $216,000/QALY (9). Furthermore, a more recent analysis by Haag and colleagues demonstrated in-home AED use in children at low to intermediate risk for SCD (0.8% risk) to be cost-effective (ICER, $86,458/QALY) (38). Our analysis is distinct from these earlier analyses, because the availability of a pocket-sized, smart-phone based AED with CPR prompts allows for access to a SMART strategy both in and out of the home. This is an important distinction, because while up to 70% of cardiac arrests occur in the home, the availability of an AED on one's person facilitates more rapid access to defibrillation, which should translate to better outcomes (9, 39).

Sudden cardiac arrest continues to be a significant societal and public health burden, and a preventable cause of death, with coronary artery disease being a leading etiology of SCA for individuals 35 years and older (5, 6, 40, 41). While ICDs may benefit those with the highest risk for SCA, the majority of deaths from cardiac arrest occur in individuals in whom ICD prophylaxis is not warranted (42). Without rapid access to defibrillation, SCA survival relies on EMS response time and availability of defibrillation, which may not arrive soon enough (29). Notably, EMS response times may exceed those modeled here, particularly in rural settings and neighborhoods characterized by higher rates of poverty (43, 44). The availability of a SMART strategy could help to optimize health equity for SCA victims who suffer from longer EMS response and could be even more cost-effective for these populations. For example, in a 2018 study Hsai et al. reported impoverished neighborhoods had 10% longer EMS response times, while a study by Peters et al. of the National Emergency Medical Services Information System demonstrated longer response times for rural vs. suburban or urban settings (7.5 vs. 5.9 min, *p* < 0.001) (43, 44). Early defibrillation and CPR are key links in the American Heart Association chain of survival; when considering outcomes of SCA seconds matter (45, 46). To address the need for rapid AED access, the United States Food and Drug Administration approved in-home AED use in 2002, and such devices have been available over-the-counter since 2004 (47). A SMART strategy evolves this concept further, making an ultra-portable defibrillator in a pocket-sized device that works with any smartphone, available in any setting.

Our analysis has several limitations. First, the evaluation of a SMART strategy was based on simulated patients, and we did not consider potential benefits of shared SMART devices. However, the use of bystander SMART integrated with smartphone technology would further improve the cost-effectiveness of a SMART approach, as a SMART device could be used within households as well as in other settings (i.e., pulsepoint.org) (48). Secondly, we did not consider improvement in quality of life (and HSU) experienced by patients and their families with the knowledge an AED is available at all times. In fact, rapid access to an AED is highly valued by patients. In one report, patients estimated a high likelihood of SCA survival with the use of an in-home AED (87–92%) (49). While the presence of an AED provides reassurance and enhances perceived control over heart disease, it is important to highlight that early access to defibrillation is not a substitute for early effective CPR and rapid access to prehospital emergency medical care (49). Whether or not a patient chooses to have a SMART device available will likely involve a patient-preference centered shared decision-making approach, because such a device may be more appropriate in some circumstances compared to others (50). The device may be better leveraged for individuals who share a home with another individual capable of providing aid, given that in-home arrests are less likely to be witnessed than those that occur in public (51, 52). Third, we assumed less than one-quarter of patients with SCA would have a shockable rhythm, although immediate access to a pocket AED at the time of arrest could increase the likelihood of a shockable rhythm (53). Notably, although older evidence suggests SCA to be associated with a shockable rhythm in up to 84% of cases (54), newer data suggests this rate may be significantly lower (23, 55). Fourth, it is possible hospital costs of SCA care could be higher than base-case estimates. Still, our model accounted for immediate SCA death prior to hospital admission as well as differential costs for those who survived to hospital discharge. Furthermore, sensitivity analyses explored hospital costs to $50,000 for survivors to discharge and $15,000 for patients who died during hospitalization. Fifth, we assumed a 44.7% overall SCA survival rate for patients with a shockable rhythm who received SMART and a 27.9% rate for patients not receiving SMART from published rates of public-access defibrillation (22). It must be acknowledged that variation exists in SCA survival, even with rapid defibrillation. Lastly, at present a pocket smart phone enabled AED is not yet commercially available; however, this device is currently under development and expected to provide rapid ultra-portable access to defibrillation for consumers in the coming years. Similar to prior published health and economic analyses which have evaluated cost-effectiveness of healthcare devices prior to commercial availability, this analysis is leveraged to inform the adoption of emerging technology (56–59). The present analysis serves to evaluate the use of not only these devices, but to more widely understand the contemporary health and economic benefits of rapid access to a personal AED.

## Conclusion

A small AED with CPR prompts for rapid treatment of SCD is a cost-effective intervention in patients at elevated SCA risk. The linkage of such a device to a smart phone has the potential to greatly improve health and economic outcomes in the United States and may prevent upwards of 100,000 SCA fatalities. Such devices could be affordable, accessible, and available in the near future and provide a significant cost-effective societal benefit in reducing cardiac mortality and morbidity.

## Data Availability Statement

The original contributions presented in the study are included in the article/supplementary materials, further inquiries can be directed to the corresponding author/s.

## Author Contributions

MSS, EG, MS, and DF contributed to the conception and design of the work and data collection. All authors contributed to the data analysis and interpretation, drafting of the article, critical revision of the article, and final approval of the work.

## Conflict of Interest

MSS has a family member who is employed by Altrix Medical, is a member of the Joint Taskforce on Allergy Practice Parameters, and serves as a member of the editorial boards of the Journal of Allergy and Clinical Immunology in Practice, the Annals of Allergy, Asthma, and Immunology, and the Journal of Food Allergy. EA is an employee of Public Health Agency of Canada (PHAC); the views expressed in this article are hers and not that of PHAC. JO declares the following: Research/Adjudication: AZ, GSK, Sanofi, Novartis; Consultant: GSK, AZ, Sanofi; Associate Editor: Annals of Allergy Asthma Immunology, AllergyWatch; Section Editor: Current Opinion of Allergy; Royalties: Up to Date; Board Liaison ABIM for ABAI. MS holds a patent related to a smart-phone enabled small AED and is employed by Altrix Medical. DF is employed by Altrix Medical. Altrix Medical's Smart AED is supported by the National Science Foundation under Grant No. 1842149 and under Cooperative Agreement No. 2026090. MG was supported by grant #5K08HS024599-02 from the Agency for Healthcare Quality and Research during the preparation of this manuscript; is an expert panel and coordinating committee member of the NIAID-sponsored Guidelines for Peanut Allergy Prevention; has served as a consultant for the Canadian Transportation Agency, Thermo Fisher, Intrommune, and Aimmune Therapeutics; is a member of physician/medical advisory boards for Aimmune Therapeutics, DBV Technologies, Sanofi/Genzyme, Genentech, Nutricia, Novatris, Kaleo Pharmaceutical, Nestle, Acquestive, Allergy Therapeutics, Pfizer, US World Meds, Allergenis, Aravax, and Monsanto; is a member of the scientific advisory council for the National Peanut Board; has received honorarium for lectures from Thermo Fisher, Before Brands, multiple state allergy societies, the ACAAI, the EAACI; is an associate editor for the Annals of Allergy, Asthma, and Immunology; and is a member of the Joint Taskforce on Allergy Practice Parameters. The remaining authors declare that the research was conducted in the absence of any commercial or financial relationships that could be construed as a potential conflict of interest.

## Publisher's Note

All claims expressed in this article are solely those of the authors and do not necessarily represent those of their affiliated organizations, or those of the publisher, the editors and the reviewers. Any product that may be evaluated in this article, or claim that may be made by its manufacturer, is not guaranteed or endorsed by the publisher.

## Abbreviations

AED: automatic external defibrillator
HP: healthcare perspective
ICD: implantable cardioverter defibrillator
ICER: incremental cost-effectiveness ratio
NMB: net monetary benefit
QALY: quality adjusted life year
SMART: small AED for rapid treatment of SCA
SCA: sudden cardiac arrest
SP: societal perspective.
